# Diagnosis and treatment of isolated celiac artery dissection following blunt trauma: A case report

**DOI:** 10.1016/j.ijscr.2021.106617

**Published:** 2021-11-19

**Authors:** Tohru Ishimine, Takahiro Ishigami, Kohei Chida, Kyohei Kawasaki, Naoki Taniguchi, Toshiho Tengan

**Affiliations:** Department of Cardiovascular Surgery, Okinawa Chubu Hospital, 281, Miyazato, Uruma-shi, Okinawa 904-2293, Japan

**Keywords:** CA, celiac artery, MVA, motor vehicle accident, CT, computed tomography, HD, hospitalization day, Celiac artery dissection, Celiac artery injury, Blunt abdominal trauma

## Abstract

**Introduction:**

Celiac artery (CA) dissection due to blunt abdominal trauma is extremely rare and, as such, the clinical features of this potentially life-threatening injury have not been clearly defined, nor have treatment strategies been established.

**Presentation of case:**

We describe the case of a 61-year-old man who presented to our emergency department after a motor vehicle accident. Although the patient did not report abdominal pain, enhanced computed tomography (CT) revealed CA dissection. The patient was treated conservatively using antiplatelet therapy and was discharged from the hospital on day 8, without complications.

**Discussion:**

As abdominal pain is not a common presenting factor of CA dissection after blunt trauma, it should be suspected as a potential injury in all affected patients and comprehensively assessed, with CT being the most useful diagnostic modality.

**Conclusion:**

In the absence of any signs of organ ischemia, changes in the CA aneurysm, and persistent, severe abdominal pain following blunt abdominal trauma, conservative treatment is indicated, with or without anticoagulation or antiplatelet therapy.

## Highlights

• Celiac artery (CA) dissection due to blunt abdominal trauma is extremely rare.
• We describe a case of CA dissection after a motor vehicle accident.
• Enhanced computed tomography revealed the CA dissection.
• The patient was treated conservatively and was discharged from hospital on day 8.

## 1 Introduction

The clinical features of celiac artery (CA) injury after blunt abdominal trauma should be clarified, and consensus-based treatment should be determined, as data are lacking in this very rare, potentially life-threatening condition. We here describe a case of CA dissection following a motor vehicle accident (MVA) that was successfully treated with antiplatelet therapy. We also discuss the literature on traumatic CA injuries. This case report was written in line with the SCARE 2020 criteria [1].

## 2 Presentation of case

**Fig. 1:**
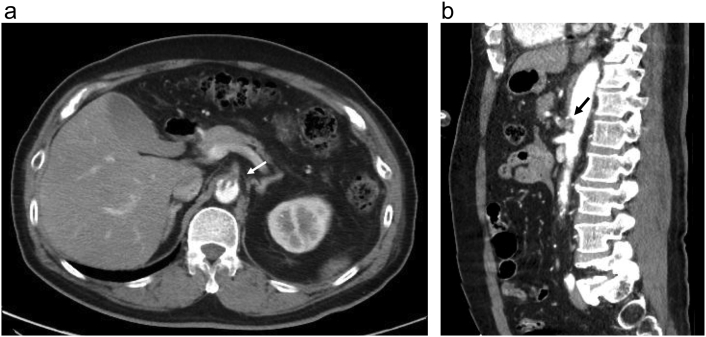
Axial (a) and sagittal (b) contrast-enhanced computed tomography images showing an intimal tear near the celiac artery origin (arrow).

A 61-year-old man was admitted to our emergency department following an MVA. The patient had a history of hypertension and hyperuricemia. On arrival, the patient was intoxicated, with a Glasgow Coma Scale score of 12 (E3, V4, and M5). At that time, he had a blood pressure of 142/66 mmHg, pulse rate of 75 beats/min, respiratory rate of 22 breaths/min, and an O_2_ saturation of 100% with an oxygen mask. The patient complained of left chest pain, but had no abdominal pain. The abdomen was soft on palpation, with normal bowel sounds and no external evidence of injury. Moreover, the focused assessment with sonography in trauma imaging of the abdomen showed no indication of injury. The laboratory results were within the respective normal limits (hemoglobin, 14.1 g/dl; platelets, 27.9 × 10^4^ platelets/μl; and creatinine, 1.01 mg/dl), except for mild leukocytosis (white blood cell count, 10.2 × 10^3^/mm^3^) and elevated liver enzymes (aspartate aminotransferase and alanine aminotransferase, 48 U/l and 31 U/l, respectively). Whole-body enhanced computed tomography (CT) imaging revealed fractures in ribs 2 to 11 on the left side, a mesenteric edema of the transverse colon with a small amount of intraperitoneal fluid indicative of possible mesenteric injury, and severe stenosis near the origin of the CA due to an intimal tear extending into the aorta (Fig. 1). The dissection did not progress into the hepatic and splenic arteries, and distal perfusion of the CA was maintained by collateral blood flow. Thus, there was no ischemia in the liver, spleen, or intestinal tract, nor brain or pulmonary contusions, and no atherosclerosis in the aorta or visceral arteries. Moreover, there was no abdominal pain, which would have been indicative of intestinal ischemia.

**Fig. 2:**
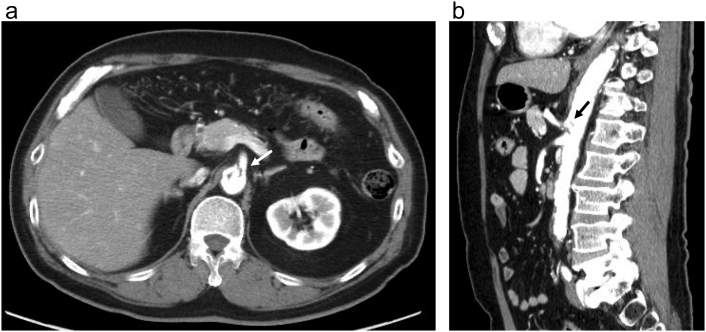
Axial (a) and sagittal (b) contrast-enhanced computed tomography images acquired 3 months after the injury showing an intimal tear shrinkage (arrow).

Antiplatelet therapy (aspirin, 100 mg) was initiated for CA stenosis treatment, and the multiple rib fractures were managed conservatively. Although we administered oxygen through a nasal cannula for 2 days, the patient's respiratory state remained stable. Therefore, neither ventilator management nor noninvasive positive-pressure ventilation were required. The patient recovered well, and abdominal pain remained absent. CT performed on hospitalization day (HD) 3 showed that the intimal tear of the CA origin had not markedly changed, and there was no organ ischemia or aneurysmal CA change. Visceral duplex sonography performed on HD 4 revealed a CA peak systolic velocity of 336 cm/s, indicative of significant stenosis. The patient was discharged on HD 8 without complications. Follow-up CT examination in the outpatient clinic 3 months after the injury revealed a decreased CA dissection size and liver enzyme level normalization (Fig. 2).

## 3 Discussion

In this report, we describe the case of a patient who presented with a CA dissection following an MVA in the absence of abdominal pain. The incidence of abdominal vascular injuries after blunt abdominal trauma is low (5%–10%) [2], with the involvement of the CA in only 1%–2% of these cases [3].

We identified 8 previous reports on CA dissection after blunt abdominal trauma [3], [4], [5], [6], [7], [8], [9], [10] (9 cases in total), the features of which are summarized and compared with the present case in Table 1. All patients were male, with a mean age of 44.4 years. CA dissection was caused by an MVA and fall in 8 (80%) and 2 (20%) cases, respectively. The survival rate was 90%, as 1 patient died due to hepatic failure secondary to progression of CA dissection on HD 7 [3]. Among the 10 cases, abdominal pain was a clinical feature in only 4 (40%) patients. Therefore, CA screening should be performed in all cases of blunt abdominal injuries.

**Table 1:** Summary of cases reports on celiac artery dissection after blunt trauma.

Author/year	Age/sex	Mechanism of injury	Abdominal pain	Treatment	Outcome
Kirchhoff (2007)	66/M	MVA	Unknown, as the patient was unconscious	Conservative management	Death on HD 7 due to fulminant hepatic failure
Suchak (2007)	41/M	MVA	(+)	Endovascular stenting	Discharged on day 5 after procedure
Gorra (2009)	29/M	Fall from height (9 m)	(−)	aAnticoagulation therapy (heparin), followed by warfarin for 3 months	Discharged on HD 4
Laeseke (2012)	47/M	Motorcycle traffic accident	(+)	Antiplatelet therapy using aspirin (81 mg)	Discharged
Sarkar (2012)	26/M	MVA	(−)	aAnticoagulation therapy using enoxaparin, followed by warfarin for 3 months	Discharged
Brown (2014)	31/M	Motorcycle traffic accident	(−)	Conservative management	Discharged on HD 10
Rosenthal (2015)	26/M	Fall from height (46 m) into a river	(−)	Antiplatelet therapy using aspirin (81 mg)	Discharged
Han (2017)	68/M	Bicycle traffic accident with a motorcycle	(+)	Antiplatelet therapy using aspirin (81 mg) for 3 months	Discharged on HD 6
	49/M	Motorcycle traffic accident	(+)	Anticoagulation therapy (heparin) for 3 days, followed by aspirin (100 mg)	Discharged on HD 4
Present case	61/M	Pedestrian struck by motor vehicle	(−)	Antiplatelet therapy using aspirin (100 mg)	Discharged on HD 8

M, male; MVA, motor vehicle accident; HD, hospital day.

a The dose of warfarin is not known.

Salim et al. reported that in high-energy trauma (such as a motor vehicle crash at >35 mph; a falling incident >15 ft; an automobile hitting a pedestrian, with the pedestrian thrown >10 ft; and being assaulted, resulting to a decreased level of consciousness), when trauma pan-scan was performed in patients with no obvious signs of trauma on the initial physical examination, abnormal findings were found in 19% (3.5% head, 5.1% cervical, 19.6% thoracic, and 7.1% abdominal) of all patients, and the treatment plan was changed [11]. In this case, the patient was asymptomatic; however, owing to the injury mechanism of being pulled by a car, a CT scan was performed. The use of contrast-enhanced CT in most cases revealed luminal filling defects, intimal flap, and surrounding hematoma diagnostic of traumatic CA dissection [9]. Although angiography is useful for evaluating collateral flow distal to the occlusion, CT is considered superior to angiography for CA dissection diagnosis as it provides superior image resolution and is less invasive.

Currently, there are no consensus guidelines for CA dissection management following blunt abdominal trauma. Treatment strategies for spontaneous CA dissection may be applicable, as it is more common than traumatic dissection. Specifically, CA dissection is conservatively treated with anticoagulant or antiplatelet therapy to prevent thrombotic occlusion of the CA. However, there is still no consensus on whether either strategy is better when conservatively treating both spontaneous and traumatic CA dissection; treatment is essentially decided based on physician preference and institutional guidelines. In fact, patients with blunt traumatic CA dissection were previously treated conservatively with antiplatelet or anticoagulant therapy, as shown in Table 1. Therefore, we chose antiplatelet treatment as it carries a reduced bleeding risk for patients with multiple trauma compared with anticoagulant treatment [5].

Endovascular stenting and open surgery, such as bypass grafting, are indicated when signs of organ ischemia, changes in the aneurysm, and persistent uncontrolled abdominal pain are observed [12]. Therefore, in our case, we considered these interventions not mandatory since the patient was in good condition and free of complications. Interestingly, endovascular stenting was required in only 1 previous case (Table 1) due to persisting severe abdominal pain [7], with successful conservative management achieved in all other cases. However, close observation of fulminant hepatic failure secondary to progressive dissection of the hepatic artery is necessary [4].

## 4 Conclusion

Overall, CA dissection is a life-threatening injury and an extremely rare complication of blunt abdominal trauma. The index of suspicion should be high in cases of such trauma, even in the absence of abdominal pain; enhanced CT is a useful diagnostic modality in this context. Although most cases of blunt traumatic CA dissection can be successfully managed conservatively, careful monitoring of these patients is required for early identification of dissection progression, which could lead to hepatic failure.

## Funding

No source of funding.

## Ethical approval

This case report was approved by the Ethics Committee of Okinawa Chubu Hospital.

## Consent

Written informed consent was obtained from the patient for publication of this case report and accompanying images. A copy of the written consent is available for review by the Editor-in-Chief of this journal on request.

## Author contributions

Tohru Ishimine: Study design, data collection, and writing the paper.

Takahiro Ishigami: Data collection and obtaining the images.

Kohei Chida: Data collection and obtaining the images.

Kyohei Kawasaki: Data collection and obtaining images.

Naoki Taniguchi: Data collection and obtaining images.

Toshiho Tengan: Reviewing the manuscript.

## Research registration

Not applicable.

## Guarantor

Tohru Ishimine.

## Provenance and peer review

Not commissioned, externally peer-reviewed.

## Declaration of competing interest

All authors declare no conflicts of interest.
